# Primary Care Physicians’ Knowledge, Attitudes, and Experience with Personal Genetic Testing

**DOI:** 10.3390/jpm9020029

**Published:** 2019-05-24

**Authors:** Susanne B. Haga, Esther Kim, Rachel A. Myers, Geoffrey S. Ginsburg

**Affiliations:** 1 Duke Center for Applied Genomics and Precision Medicine, Duke University School of Medicine, Durham, NC 27708, USA; rachel.myers@duke.edu (R.A.M.); geoffrey.ginsburg@duke.edu (G.S.G.); 2 23andMe, Inc., 899 W Evelyn Ave, Mountain View, CA 94041, USA; ekim@23andme.com

**Keywords:** genomics, education, primary care

## Abstract

Primary care providers (PCPs) will play an important role in precision medicine. However, their lack of training and knowledge about genetics and genomics may limit their ability to advise patients or interpret or utilize test results. We evaluated PCPs’ awareness of the role of genetics/genomics in health, knowledge about key concepts in genomic medicine, perception/attitudes towards direct-to-consumer (DTC) genetic testing, and their level of confidence/comfort in discussing testing with patients prior to and after undergoing DTC testing through the 23andMe Health + Ancestry Service. A total of 130 PCPs completed the study. Sixty-three percent were board-certified in family practice, 32% graduated between 1991 and 2000, and 88% had heard of 23andMe prior to the study. Seventy-two percent decided to participate in the study to gain a better understanding about testing. At baseline, 23% of respondents indicated comfort discussing genetics as a risk factor for common diseases, increasing to 59% after undergoing personal genetic testing (PGT) (*p* < 0.01). In summary, we find that undergoing PGT augments physicians’ confidence, comfort, and interest in DTC testing.

## 1. Introduction

Genetic and genomic testing is expanding across medical specialties and increasingly ordered and managed by non-geneticists [1]. In addition to the breadth of tests, access to testing is evolving beyond the traditional route of health provider-ordered clinical testing. Mechanisms for accessing genetic data include consumer-ordered tests through direct-to-consumer (DTC) companies without medical authorization, consumer-initiated tests ordered by the company’s network of affiliated clinical providers, and research studies that return personal genetics/genomics information to study participants.

Regardless of delivery model, patients may share and seek advice about their genetic results with a health provider [2,3,4], presumably a primary care provider (PCP) with whom they are most likely to seek medical advice from initially. Appropriate test utilization and patient engagement will depend on provider awareness and knowledge about genomic applications. Despite reported consumer enthusiasm for genetic testing and national initiatives focusing on precision medicine, several studies have reported that knowledge among healthcare providers, including PCPs, is lacking [5,6,7,8,9,10,11,12]. Exposure to genetics/genomics or precision medicine in medical curricula is also limited to the initial years, and then dropping precipitously [13]. For practicing providers, opportunities to engage with and learn about genomic medicine may be limited. Thus, identifying effective strategies for provider education is critical to close the gap in physician preparedness for genetics/genomics and precision medicine. 

Participatory, active, or experiential learning approaches utilizing personal genetic testing (PGT) enable providers and other learners to gain first-hand knowledge about genetic testing, to have an opportunity to review their raw data and test results, and to learn how to interpret and apply them to preventive care and treatment decisions. Health provider experience with PGT as an educational approach has been reported to enhance the understanding of clinically relevant concepts in genomics, confidence to respond to genetics information in clinical settings, and attitudes about testing and is offered at many schools and professional meetings [14,15,16]. While some programs provide in-house testing to their students or health providers [17], others have utilized commercial services (Illumina UYG) or DTC genetic testing services (23andMe Health + Ancestry Service) [18,19]. However, many of these studies have been conducted in small populations, in structured educational programs, or in single academic settings, limiting the generalizability of the data.

In this paper, we report the findings of a national study evaluating the impact of self-directed, participatory learning utilizing a DTC genetic testing service for a self-selected group of practicing PCPs on their knowledge, attitudes, and preparedness related to genomic medicine and DTC genetic testing.

## 2. Materials and Methods

*Survey Development*. The surveys were developed via a collaboration between Duke University School of Medicine, 23andMe, Inc., and SoundRocket. The pre-test (baseline) survey consisted of 37 questions regarding physician demographics, education and experience with genetic testing, factual knowledge about genetic variation [20], perceived knowledge of different areas of genomics such as genetics of complex disease and pharmacogenetics [5], and attitudes toward DTC testing. The questions regarding genetic variation were from the genetic variation knowledge assessment index developed and validated by Bonham et al. [20]. The post-testing (follow-up) survey consisted of 35 questions, including the same pre-test survey questions regarding perceived and factual knowledge about genetic variation and genomics and attitudes toward DTC testing as well as questions about their personal experience reviewing the genetic test results. The surveys were designed as a self-administered, interactive, web-based survey with an estimated 15-min completion time. The first four questions in the pre-test survey screened respondents for eligibility; respondents were deemed ineligible for the study if they had prior experience with 23andMe, practiced fewer than 20 h/week, or were not board-certified in Family Practice or Internal Medicine. The surveys were administered as on-line web surveys, accessible via mobile devices and tablets, desktop or laptop computers. Apart from the screening questions and consent, respondents could skip any question(s) they did not wish to answer.

*Provider Population*. The eligible population of physicians for this survey were identified by an email campaign facilitated by IQVIA, a health data science and clinical research company with access to members of the health care community. IQVIA maintains a large database of active physicians in the United States. To gauge interest in the study, IQVIA emailed a brief description of the Physician Personal Genetic Test Study, along with an invitation to share personal contact information for those wishing to be considered to participate in the study. To help ensure the highest percentage of eligible respondents possible, IQVIA filtered their database and selected physicians who met the following criteria: (1) specialty/physician type as internal medicine or family practitioner, and (2) medical school graduation year between 1950 and 2017. To help ensure gender representativeness, IQVIA drew samples of males and females proportional to the records in their database. Finally, any physicians who had specifically requested no contacts from 23andMe, Duke, and SoundRocket were suppressed in the IQVIA database and could not be selected. 

*Data Collection.* A study invitation was deployed in two email recruits by IQVIA, each consisting of 30,000 emails. The first email was released on June 1–June 2, 2017; a second group of emails was released on July 18, 2017. An email invitation to the pre-test survey (which also included the screening questions) was then sent to all physicians who indicated potential interest in participating in the study. Respondents who completed both surveys also received a $25 Amazon gift card.

*Testing.* Physicians who completed the pre-test survey were provided with a link to the 23andMe website; the link was auto-authenticated to a 23andMe online shopping cart where physicians could order the Health + Ancestry Service at no cost as part of the study (a $199 value). All respondents were given access to pre-purchase information available to all consumers, including FDA labeling, before completing the order. Respondents were sent a test kit including a saliva sample collection vial and prepaid return envelope. After their saliva sample was received by the laboratory and analysis testing was completed, respondents were notified that their personal reports were available via a secure online portal. Respondents had the option to review all or any part of their 23andMe Reports and had access to other tools available at 23andme.com, subject to the terms of the Web Agreements (specifically, the 23andMe Terms of Service). Respondents who completed both the pre- and post-testing surveys were granted unlimited access, without expiration, to their 23andMe portal.

The 23andMe website (23andme.com) provides background information about each testing category (Ancestry, Carrier Status, Genetic Health Risk, Wellness, and Traits), sample reports, background on genetics and genomics, and the testing process. 

*Data analysis.* The analysis was based on survey data from those who completed both the pre-testing survey and post-testing survey. Summary counts and percentages were calculated for all questions. Differences in participant features (demographics, attitude, and knowledge) between those who completed the study and those who did not complete were tested using Pearson’s chi-squared test. Changes in response from pre to post-test questions were assessed using McNemar’s Chi-squared test. Variation in responses due to either graduation year or practice type was assessed using Pearson’s chi-squared test. All analyses were conducted using the statistical program R. 

## 3. Results

### 3.1. Respondent Characteristics

A total of 450 physicians responded to the initial recruitment email; 342 consented to participate, and based on the screener, 219 were eligible to participate. A total of 203 completed the baseline survey. Respondents who completed the baseline survey were sent a waiver code for 23andMe Health + Ancestry Service; 78% (159) submitted a sample for testing by 23andMe; two samples contained insufficient genetic material for testing, yielding a total of 157 respondents that completed testing and received their genetic reports. A total of 130 respondents completed the post-testing survey. No significant differences with respect to respondent demographics, knowledge, or attitudes were observed between respondents who completed only the baseline survey and those who completed both baseline and post-testing survey except for race (*p* = 0.05; respondents that went on to complete the post-testing survey were less racially diverse). Based on the 130 respondents that completed both the pre-testing survey and the post-testing survey, 63% of respondents were board-certified in family practice, 33% graduated in 2001 or later, 87% were White, and 51% were male (Table 1).

### 3.2. Education about Genomic Medicine & Experience with Genetic Medicine

Overall, the majority of respondents (62%) indicated that they did not receive any type of formal education in genomic medicine or using genetic information to make individualized risk predictions and treatments decisions. Those who had some formal education or training received it primarily in medical school (25%). Formal education in genomic medicine was associated with recency of graduation (divided before or after 1991) (*p* = 0.002). In the past year, 76% of respondents indicated that they had not participated in any informal genomic medicine education such as continuing medical education or conferences; no association with informal learning and recency of graduation was observed. Respondents indicated that their preferred mode of education for genomic medicine is online CME programs (42%), followed by professional meetings (21%), and in-person CME such as grand rounds (18%).

Forty-two percent of respondents indicated that they always or most of the time collected three-generation family histories (26% rarely or never did). Respondents with some formal education in genomic medicine trended toward more likely to collect a family history (*p* = 0.05). Fifty-two percent of respondents indicated they had referred one to three patients for a genetic consultation in the past year (28% had not referred any patients in the past year). Forty-four percent of respondents indicated that they have never ordered a genetic test, while 34% had ordered 1 to 3 genetic tests in the past year. No significant association between year of graduation or specialty was observed with frequency of test ordering. Of the respondents that had ordered a genetic test in the past year, pre-symptomatic or susceptibility testing was the most common type of genetic test ordered (56%), followed by carrier testing (48%) and diagnostic testing (43%). Single gene tests were mostly ordered (44%) compared to gene panels (29%) or karyotypes (14%). 

Regarding respondents’ comfort level with discussing risk factors associated with common diseases, in the pre-testing phase, most respondents indicated they felt very comfortable (a rating of 4 or 5 on a 5-point scale) discussing most factors except genetics (Table 2).

### 3.3. Perception and Attitudes about DTC Testing

The majority of respondents had heard of 23andMe prior to the study (88%); no significant association was observed between graduation year (before/after 1991). Respondents who had heard of 23andMe prior to this study were significantly more likely to complete the study (*p* = 0.003). The top motivating factors for participating in the study were to gain a better understanding about clinical utility and application of genetic testing (72%) and to gain hands-on learning to better counsel patients (70%). Other top motivating factors included contributing to advancement of science (57%), learning about themselves (52%), and understanding what their patients are experiencing (51%). 

Overall, attitudes improved significantly following testing regarding confidence in discussing results of DTC genetic testing, knowledge about discussing risks, benefits and results of DTC genetic testing as well as patients’ ability to understand their results and perceived benefit (Table 3). At baseline (pre-testing), 34% of respondents strongly or somewhat agreed with the statement that they would recommend DTC testing to their patients who inquired about it; post-testing, a significantly higher proportion of respondents strongly or somewhat agreed with that statement (62%; *p* < 0.00001). No significant change in attitudes was observed regarding the important role of DTC testing in their practice nor regarding patient interest in DTC testing. 

At baseline, the most commonly indicated benefits of DTC testing and personal genetic information services were as a tool for motivating a healthy lifestyle (73%), early detection and management of adult-onset inherited diseases (70%), and incentivizing individuals to actively participate in their health care (70%). These top three perceived benefits remained the same at post-testing. 

At baseline, the top three concerns with DTC personal genetic information were the lack of established clinical practice guidelines (72%), uncertain clinical utility (65%), and personal lack of knowledge to interpret the information (56%). Post-testing, their top three concerns were uncertain clinical utility (68%), potential for patient anxiety (62%), and lack of established clinical practice guidelines (62%). 

### 3.4. Perceived and Actual Knowledge of Key Concepts in Genetics and Genomics

We asked about respondents’ perceived and actual knowledge of genetics and genomics concepts, specifically investigating perceived and actual familiarity with the following areas: DTC genetics, pharmacogenetics, the genetics of complex disease, genome-wide association studies (GWAS), basic genetics principles, and when and how to incorporate genomic information into practice. Pre-testing, 27% of respondents strongly or somewhat agreed with the statement that they have sufficient knowledge about DTC genetic testing to help patients understand their results, almost tripling to 73% post-testing (*p* < 0.01) (Table 3). In contrast, 12% strongly or somewhat agreed with the statement that most physicians have sufficient knowledge to help patients understand the results of DTC genetic tests on the pre-test, which increased to 47% post-testing (*p* < 0.01). Respondents’ perceived understanding of the limitations, benefits and risks of DTC genetic testing doubled from 44% pre-testing to 84% post-testing (*p* < 0.01). Pre-testing, 20% strongly or somewhat agreed with the statement that they felt confident that they could discuss the results of DTC genetic testing with their patients, increasing to 71% post-testing (*p* < 0.01). Pre-testing, 9.3% felt that most of their patients could understand the results, increasing to 38% post-testing (*p* < 0.01).

Respondents were asked to indicate their level of knowledge in five areas of genetics and genomics in the pre-test survey. Overall, almost all respondents rated their knowledge of GWAS as none or minimal (92%) (Table 4). In contrast, the majority rated their knowledge of basic genetics principles at a moderate to expert level of understanding (90%). Sixty-one percent had no or minimal knowledge about when and how to integrate genomic medicine information into practice. No differences by specialty or year of graduation were observed.

Respondents were asked to answer seven true/false knowledge questions in five areas of genetics and genomics in the pre- and post-test surveys. We excluded one question from the analysis due to ambiguity in the wording of the question. The majority of respondents answered five out of six questions correctly pre- and post-testing (Table 5). The question with the lowest score was about the percentage of genome identity. No significant difference in knowledge was observed between pre-testing and post-testing for any questions.

### 3.5. Primary Care Physicians’ Personal Experience with Personal Genomic Testing

Five categories of reports, Ancestry, Carrier Status, Genetic Health Risk, Wellness, and Traits, were available at the time (Appendix A). Of the 130 respondents, two reported that they did not specifically review any reports. Ancestry and Genetic Health Risk reports were reviewed most, 96% and 89%, respectively (Table 6). A total of 54% reported spending more than 20 min reviewing their results. A total of 58% reported accessing their 23andMe reports two to three times, and 23% reported accessing them four or more times. Almost half (46%) indicated it was very easy to review and understand results based on how the results were presented (38% indicated somewhat easy). A total of 28% (of 121 reviewing reports) indicated that the health-related reports were somewhat or very surprising (either unknown or conflicted with current understanding of family history). With respect to the ancestry reports, 48% (of 124 reviewing the report) indicated that their results were somewhat or very surprising (either unknown or conflicted with current understanding of their family’s roots).

A total of 61% suggested that their experience with PGT would be improved if more diseases were tested for. Most respondents (75%) did not use or consult any additional resources to better understand their test results. Ten percent of respondents looked up a particular disease through a general online search query and 12% looked up the published literature on a particular disease or gene/genetic variant. None reported consulting with a genetic counselor. 

### 3.6. Impact of PGT on PCPs’ on Comfort/Confidence, Perceived Knowledge, Interest, and Perceived Value

Fifty-nine percent of respondents reported that the testing experience improved their knowledge of genomic medicine a little, 12% reported that it improved their knowledge greatly, and 28% reported no impact. Post-testing, a significantly greater proportion of respondents indicated they felt very comfortable discussing the role of patient’s health status (*p* = 0.04), genetics (*p* < 0.000001), and disease risk race/ethnicity (*p* = 0.02) related to(Table 2). With respect to the impact of the testing experience on their interest in genomic medicine, 56% reported that their interest increased a little and 30% reported that their interest increased greatly. Fifty-three percent of respondents indicated that they anticipated participating in 1–2 educational activities (e.g., CMEs, conferences, research) associated with genomic medicine in the next 12 months (24% had participated in such educational events in the past year); 42% indicated that they did not anticipate participating in any such educational activities. Sixty-one percent of respondents indicated that they would somewhat or very likely recommend PGT to their colleagues as a way to learn more about genomic medicine.

## 4. Discussion

The importance of provider education has been at the forefront of discussions about workforce readiness, appropriate test utilization, and patient engagement. In particular, PCPs will likely be one of the major groups that will be integrating genomic information to inform patient risk assessment, prevention strategies, and treatment decisions. In our study, we found that by increasing PCP familiarity with PGT through participatory learning, several positive changes in attitudes and comfort/confidence in genomic medicine were reported. Specifically, after testing, PCPs demonstrated higher interest toward DTC genetic testing, likelihood of recommending testing to interested patients, perceived adequacy of knowledge to help patients understand results, and intention to participate in educational activities (e.g., CMEs, conferences, research) about genomic medicine in the next 12 months. With the rapidly evolving genetic testing landscape and knowledge base regarding disease susceptibility and medication response, it is essential that providers stay current to ensure appropriate use of testing.

The interest in and effectiveness of participatory or experiential learning has been demonstrated for learners at multiple levels and in several settings [19,21,22,23,24]. A positive experience with a novel application or service may improve future knowledge acquisition regarding this specific test and related applications, as well as potentially alter practice behaviors (e.g., incorporating testing into practice). Although attitudes and PCPs’ comfort level with offering this type of testing improved, we did not observe a significant impact on PCPs’ knowledge about genetics in our study (although this was high for most questions). This may be due to limited knowledge assessment tools and/or limited time spent reviewing the test reports, and/or lack of motivation to seek additional information. The learning experience was not guided and therefore, key concepts were not flagged for PCPs to review. Thus, PCPs may benefit from additional learning opportunities such as guided, short online modules on various aspects of genomic medicine in combination with or following participatory learning strategies to enhance their knowledge in specific areas. Though we are unaware of respondents’ scope or depth of learning about genetics, genomics, and DTC testing from 23andMe’s website and various other resources during this study, which may have influenced their responses and overall experience, this singular PGT testing experience is likely not sufficient enough training. With the rapid changes in testing and evidence, continuous education is needed to minimize errors in patient counseling or medical mismanagement.

The lack of reported impact on PCPs’ attitude about the importance of DTC genetic testing in their practice may be due to the limited number of health reports available at the time of study. During the study period, only seven health predisposition reports were available, but currently (April 2019), eleven reports are available. Interestingly, the finding that significantly more participants thought that most of their physician colleagues would have sufficient knowledge to help patients and most of their patients could understand their DTC genetic test results after the intervention. We speculate that unfamiliarity with DTC and limited knowledge and training in genetics may have inflated respondents’ perceptions about the difficulty of the reports. The test provides both health and non-health related information, but we did not probe respondents’ perceptions or attitudes of the different types of information. 

Online educational programs were the preferred mode of learning indicated by respondents. Digital-based educational opportunities or e-learning are convenient, easily updated, and have a large outreach. For example, point-of-care learning, or clinical decision support systems have been developed and implemented to provide clinicians with information about pharmacogenetic testing and prescribed medications [25,26,27], support care for patients affected with genetic conditions [28], referral for genetic counseling [29], and clinical recommendations for family health history (MeTree) at the time of care [30]. Several genetics and genomics e-learning programs have been developed for a wide range of health providers, but only some have been shown to be effective with respect to both knowledge and impacted behaviors [31,32,33]. Laboratory reports also increasingly include detailed information regarding interpretation, follow-up care, and clinical guidelines related to the test result. However, the shift from passive to interactive learning strategies may enable greater learning and behavior change with complex topics such as genomics/genetics and precision medicine.

Some limitations of this study should be noted. The study had a low response rate, and potentially biased toward those familiar with 23andMe and PGT. Thus, study respondents may not represent the primary care provider population and the results may not be generalizable. We assessed the effectiveness of this learning approach through perceived value and knowledge rather than actual knowledge or behavior change, due to the lack of a validated knowledge tool for PCPs and our inability to evaluate the impact of the learning experience on PCP behavior, respectively. In addition, the knowledge questions related to general genetic principles, and there were no questions regarding integrating genomic information into practice. We did not include questions about PCPs’ ability to interpret test results or conduct patient counseling. Lastly, respondents may have sought out information about genetics, genomics, and DTC testing from 23andMe’s website and various other resources that may have influenced their responses and overall experience. Despite the gradual movement of genetics and genomics toward primary care, PCPs have limited exposure to genetics and genomics in their formal training and the subject may not yet be prioritized highly, competing against many other areas that PCPs encounter more often [34]. With the lack of experience with traditional clinician-ordered genetic testing for risk management or diagnosis, the introduction of DTC genetic testing may further present challenges to PCPs to appropriately engage with their patients about the results, particularly given the suite of testing services included in the package. Consumers of DTC may choose to order testing for a specific reason or general interest or curiosity. Thus, the need to develop effective educational strategies to prepare PCPs to appropriately utilize and integrate genomic information into practice is critical [35]. This study demonstrates that participatory learning may be an effective mechanism to engage interested learners about this application. However, additional strategies are clearly needed to complement this approach and promote continued learning and/or to introduce PGT to PCPs with no familiarity prior to launching participatory learning-based programs. Thus, this may be an effective and time-efficient strategy to augment traditional and point-of-care approaches to improving PCPs’ knowledge, interest, and preparedness to embrace genetics/genomics and precision medicine and stimulate continued learning. Identifying provider education strategies will be crucial to increasing appropriate PGT utilization and interpretation of test results.

## Figures and Tables

**Table 1:** Characteristics of study respondents.

Characteristic	Completed both Baseline (pre-Testing) and Post-Testing Survey *n* = 130
Family Practice	82 (63.1%)
Internal Medicine	48 (36.9%)
Male	66 (50.8%)
**Race (select all that apply)**	
African-American	5 (3.8%)
Asian	6 (4.6%)
White	113 (86.9%)
Other/Multi-racial	6 (4.6%)
Hispanic/Latino	12 (9.2%)
**Year of Medical School Graduation**	
Before 1981	10 (7.7%)
1981–1990	36 (27.7%)
1991–2000	41 (31.5%)
2001 and later	43 (33.1%)
**Practice Setting Type**	
Single provider practice	13 (10.0%)
Multi-provider practice	101 (77.7%)
Hospitalist	8 (6.2%)
Other	8 (6.2%)

**Table 2:** Percentage of respondents who indicated 4 or 5 on a 5-point Scale (1 = least comfortable; 5 = most comfortable) regarding comfort level in discussing various types of risk factors for common disease with patients (*n* = 130).

	Pre-Testing	Post-Testing	McNemar’s Chi-sq Test *p*-Value
Genetics	23.1%	58.9%	<0.000001
Environmental	91.5%	94.6%	0.39
Race/ethnicity	61.5%	73.8%	0.02
Health Status	88.5%	95.4%	0.04

**Table 3:** Percentage of responses in agreement (strongly or somewhat agree) with statements about the use of direct-to-consumer (DTC) genetic testing, physician preparedness, and patient interest (*n* = 130).

Attitude	Pre-Testing (Somewhat/Strongly Agree) *n* = 130	Post-testing (Somewhat/Strongly Agree) *n* = 130	McNemar Test of Independence (*p*-Value)
I understand the limitations, risks and benefits of using DTC testing services	43.9%	83.7%	<0.01
I have enough knowledge to help patients understand the results of DTC genetic tests	26.9%	73.1%	<0.01
I feel confident about discussing DTC genetic testing with my patients.	20.0%	70.7%	<0.01
Most physicians have sufficient knowledge to help patients understand the results of DTC genetic tests	12.3%	46.9%	<0.01
If a patient of mine inquired about DTC testing, I would likely recommend that they consider it.	33.8%	62.3%	<0.01
Most of my patients could understand their DTC genetic test results.	9.3%	37.5%	<0.01
DTC genetic testing will likely play an important role in my practice	31.0%	30.0%	*p* = 1.0
Some of my patients would be interested in DTC genetic testing	86.0%	84.5%	0.81

**Table 4:** Perceived knowledge at pre-testing phase among respondents who completed the pre-testing and post-testing survey (*n* = 130).

	Pharmaco-Genetics (*n* = 130)	Genetics of Complex Disease (*n* = 130)	GWAS (*n* = 130)	Basic Genetics Principles (*n* = 130)	When & How to Incorporate Genomic Information into Practice (*n* = 130)
No knowledge	7.7%	6.9%	52.3%	0.8%	10.0%
Minimal	53.8%	48.5%	40.0%	9.2%	51.5%
Moderate	30.8%	39.2%	6.9%	58.5%	33.8%
Above Average	7.7%	5.4%	0.8%	29.2%	4.6%
Expert	0	0	0	2.3%	0

**Table 5:** Factual knowledge among respondents who completed the pre and post-testing survey (*n* = 130).

Question	Pre-Testing % Correctly Answered	Post-Testing % Correctly Answered
The DNA sequences of two randomly selected healthy individuals of the same sex are 90–95% identical. (FALSE)	31.5%	32.3%
Most common diseases, such as diabetes and heart disease, are caused by a single gene variant. (FALSE)	96.2%	97.0%
All the genetic variation in an individual can be attributed to either spontaneous (i.e., de novo) or inherited changes in the human genome. (TRUE)	63.6%	61.2%
Individual genetic variants are usually highly predictive of the manifestation of common disease. (FALSE)	77.7%	76.2%
Prevalence of many Mendelian diseases differs by racial groups. (TRUE)	86.0%	85.4%
A patient who is found to be at increased genetic risk can reduce or modify their overall disease risk with changes to their health management, treatment, or lifestyle. (TRUE)	97.0%	99.2%

**Table 6:** 23andMe reports accessed by respondents.

Type of 23andMe Reports	PCPs Who Reviewed Their 23andMe Reports	%
Ancestry (5 reports were available)	125	96.2%
Health Predisposition-Genetic Health Risk (7 reports were available)	116	89.2%
Carrier Status (43 reports were available)	115	88.5%
Traits (22 reports were available)	104	80.0%
Wellness (8 reports were available)	100	76.9%
“I do not recall which sections I reviewed”	1	0.77%
Did not review any reports	2	1.5%

## Appendix A

A list of reports available during the study period (August–November 2017).

**Health Predisposition-Genetic Health Risk reports:**

1. Age-Related Macular Degeneration
2. Alpha-1 Antitrypsin Deficiency
3. Celiac Disease
4. Hereditary Hemochromatosis (HFE-Related)
5. Hereditary Thrombophilia
6. Late-Onset Alzheimer’s Disease
7. Parkinson’s Disease

**Carrier Status reports:**

1. ARSACS
2. Agenesis of the Corpus Callosum with Peripheral Neuropathy
3. Autosomal Recessive Polycystic Kidney Disease
4. Beta Thalassemia and Related Hemoglobinopathies
5. Bloom Syndrome
6. Canavan Disease
7. Congenital Disorder of Glycosylation Type 1a (PMM2-CDG)
8. Cystic Fibrosis
9. D-Bifunctional Protein Deficiency
10. Dihydrolipoamide Dehydrogenase Deficiency
11. Familial Dysautonomia
12. Familial Hyperinsulinism (ABCC8-Related)
13. Fanconi Anemia Group C
14. GRACILE Syndrome
15. Gaucher Disease Type 1
16. Glycogen Storage Disease Type Ia
17. Glycogen Storage Disease Type Ib
18. Hereditary Fructose Intolerance
19. Herlitz Junctional Epidermolysis Bullosa (LAMB3-Related)
20. Leigh Syndrome, French Canadian Type
21. Limb-Girdle Muscular Dystrophy Type 2D
22. Limb-Girdle Muscular Dystrophy Type 2E
23. Limb-Girdle Muscular Dystrophy Type 2I
24. MCAD Deficiency
25. Maple Syrup Urine Disease Type 1B
26. Mucolipidosis Type IV
27. Neuronal Ceroid Lipofuscinosis (CLN5-Related)
28. Neuronal Ceroid Lipofuscinosis (PPT1-Related)
29. Niemann-Pick Disease Type A
30. Nijmegen Breakage Syndrome
31. Nonsyndromic Hearing Loss and Deafness, DFNB1 (GJB2-Related)
32. Pendred Syndrome and DFNB4 Hearing Loss (SLC26A4-Related)
33. Phenylketonuria and Related Disorders
34. Primary Hyperoxaluria Type 2
35. Rhizomelic Chondrodysplasia Punctata Type 1
36. Salla Disease
37. Sickle Cell Anemia
38. Sjögren-Larsson Syndrome
39. Tay-Sachs Disease
40. Tyrosinemia Type I
41. Usher Syndrome Type 1F
42. Usher Syndrome Type 3A
43. Zellweger Syndrome Spectrum (PEX1-Related)

**Wellness reports:**

1. Alcohol Flush Reaction
2. Caffeine Consumption
3. Deep Sleep
4. Genetic Weight
5. Lactose Intolerance
6. Muscle Composition
7. Saturated Fat and Weight
8. Sleep Movement

**Traits reports:**

1. Asparagus Odor Detection
2. Back Hair (available for men only)
3. Bald Spot (available for men only)
4. Bitter Taste
5. Cheek Dimples
6. Cleft Chin
7. Earlobe Type
8. Early Hair Loss (available for men only)
9. Earwax Type
10. Eye Color
11. Finger Length Ratio
12. Freckles
13. Hair Thickness
14. Light or Dark Hair
15. Newborn Hair
16. Photic Sneeze Reflex
17. Red Hair
18. Skin Pigmentation
19. Sweet vs. Salty
20. Toe Length Ratio
21. Unibrow
22. Widow’s Peak

**Ancestry**

1. Ancestry Composition
2. Your DNA Family
3. Neanderthal Ancestry
4. Maternal Haplogroup
5. Paternal Haplogroup
